# Supplementary material for: Memory-like states created by the first ethanol experience are encoded into the Drosophila mushroom body learning and memory circuitry in an ethanol-specific manner
Source: PLoS Genet. 2025 Feb 3;21(2):e1011582. doi: 10.1371/journal.pgen.1011582 (PMC11801723; doi:10.1371/journal.pgen.1011582)
Supplement: S1 Table — (DOCX) [file pgen.1011582.s006.docx]

| **Strain Name** | **Figure Alias** | **Source** | **Source ID** | **FlyBase ID (genetic)** | **Flybase ID (molecular)** |
| --- | --- | --- | --- | --- | --- |
| *amn^1^* | *amn^1^* | BDSC | 5954 | FBst0005954 | FBal0000500 |
| *rut^1^* | *rut^1^* | BDSC | 9404 | FBst0009404 | FBal0014878 |
| *Sirt1^2A-7-11^* | *Sirt1^–^* | BDSC | 8838 | FBst0008838 | FBal0181537 |
| *17d-Gal4* | *MB(αβ)^17d^* | Ulrike Heberlein | 51631 | FBti0012447 | FBal0094031 |
| *201y-Gal4* | *MB(αβγ)^201y^* | Ulrike Heberlein | 4440 | FBti0002924 | FBal0048768 |
| *5015-Gal4* | *DPM^2721^* | BDSC | 2721 | FBst0002721 | FBti0001256 |
| *Elav(c155)-Gal4* | *elav* | BDSC | 8579 | FBti0002575 | FBal0047071 |
| *GH146-Gal4* | *APL^GH146^* | BDSC | 30026 | FBti0016783 | FBal0218122 |
| *nSyb-Gal4* | *nSyb* | Julie Simpson | n/a |  |  |
| *R11D09-Gal4* | *MB(γ)^R11D09^* | BDSC | 48456 | FBst0048456 | FBti0132966 |
| *R13F02-Gal4* | *MB(all)^R13F02^* | BDSC | 48571 | FBst0048571 | FBti0133166 |
| *R16A06-Gal4* | *MB(γ)^R16A06^* | BDSC | 48709 | FBti0133387 | FBtp0057846 |
| *R19B03-Gal4* | *MB(all)^R19B03^* | BDSC | 49830 | FBti0133663 | FBtp0058122 |
| *R28H05-Gal4* | *MB(αβ)^R28H05^* | BDSC | 49472 | FBst0049472 | FBti0134561 |
| *R35B12-Gal4* | *MB(α’β’)^R35B12^* | BDSC | 49822 | FBst0049822 | FBti0135116 |
| *VT43924-Gal4* | *APL^VT^* | VDRC | 201194 | FBst0488255 | FBti0170491 |
| *VT64246-Gal4* | *DPM^VT^* | VDRC | 204311 | FBst0490696 | FBti0169413 |
| *Gad1-Gal80* | *Gad1-Gal80* | Tim Lebestky | n/a |  |  |
| *tub-Gal80^ts^* | *Gal80^ts^* | BDSC | 7019 | FBti0027796 | FBtp0017264 |
| *UAS-amn.IR* | *amn.IR* | VDRC | 5606 | FBst0470060 | FBti0081240 |
| *UAS-CaMKII.IR* | *CaMKII.IR* | BDSC | 35330 | FBst0035330 | FBti0144323 |
| *UAS-Creb2b* | *CREB2b* | BDSC | 7219 | FBti0038039 | FBtp0007906 |
| *UAS-Ddc.IR* | *Ddc.IR* | BDSC | 27030 | FBti0115397 | FBtp0040789 |
| *UAS-dsNR1.IR* | *Nmdar1.IR* | Chia-Lin Wu | n/a | n/a | FBal0268132 |
| *UAS-inx6.IR* | *inx6.IR* | VDRC | 8638 | FBtp0031064 | FBsf0000074143 |
| *UAS-inx7.IR* | *inx7.IR* | VDRC | 103256 | FBtp0049343 | FBsf0000094371 |
| *UAS-Gad1.IR* | *Gad1.IR* | BDSC | 28079 | FBst0028079 | FBti0128065 |
| *UAS-rsh.IR* | *rsh.IR^39932^* | VDRC | 39932 | FBst0463294 | FBti0097962 |
| *UAS-rsh.IR* | *rsh.IR^36758^* | BDSC | 36758 | FBst0036758 | FBti0146805 |
| *UAS-rsh.IR* | *rsh.IR^57574^* | BDSC | 57574 | FBst0057574 | FBti0163824 |
| *UAS-Rdl.IR 8-10G* | *Rdl.IR* | Ronald Davis | 89903 | FBal0243638 | FBtp0053459 |
| *UAS-Shibire^ts^* | *Shi^ts^* | BDSC | 66599 | FBti0185291 | FBtp0093850 |
| *UAS-Sirt1.IR* | *Sirt1.IR* | BDSC | 32481 | FBti0132175 | FBtp0056584 |
| *UAS-Tbh.IR* | *Tbh.IR* | BDSC | 27667 | FBst0027667 | FBti0128848 |
| *UAS-CaMKII^T287D^* | *CaMKII^T287D^* | Michael Crickmore | n/a | n/a | FBal0376580 |
| *UAS-Kay.DN* | *kay.DN* | BDSC | 7215 | FBti0038043 | FBtp0007879 |
| *UAS-Mef2.EnR* | *Mef2.EnR* | Justin Blau |  |  |  |
| *UAS-RicinA^cs^* | *Ricin^cs^* | BDSC | 38624 | FBst0038624 | FBti0147578 |
