# Supplementary material for: Memory-like states created by the first ethanol experience are encoded into the Drosophila mushroom body learning and memory circuitry in an ethanol-specific manner
Source: PLoS Genet. 2025 Feb 3;21(2):e1011582. doi: 10.1371/journal.pgen.1011582 (PMC11801723; doi:10.1371/journal.pgen.1011582)
Supplement: Attachment — Submitted filename: PGENETICS-D-24-01147 PLoS Genetics response to reviewers.docx [file pgen.1011582.s009.docx]

**Reviewers' comments:**

**Reviewer #1:** Larnerd et al., describe in their manuscript ”memory-like states created by the first alcohol experience are encoded into the Drosophila mushroom body learning and memory circuitry an alcohol-specific manner” whether the formation of rapid and chronic tolerance is sensitive to a cold shock and what kind of neurons might regulate cold shock sensitive rapid tolerance, cold chock insensitive rapid tolerance or chronic tolerance.

It is important to classify the mechanisms underlying rapid and chronic tolerance into anesthesia-sensitive and anesthesia-resistant mechanisms. It is also important to map neurons that contribute to rapid and chronic tolerance. In addition, Larnerd et al. have begun to address whether similar molecules underlying the effects of ethanol at the cellular level also contribute to rapid and chronic tolerance in the neurons identified.

What is misleading is the direct comparison to mechanisms underlying aversive associative learning and memory.

First, the effect of ethanol is doses dependent. We do not know whether the SD50 of the first ethanol exposure in flies is evaluated as negative or positive. Positive and negative effects might contribute to the development of tolerance. The ethanol exposure might result in a mixture of effects- either positive or negative.

Second, the endpoint of E1 (is described in the material and method as “between 12 to 20 min”) is very unprecise. If the endpoint was fixed, the question arises whether the effect for the flies is either negative or positive. That will influence whether the mechanism of rapid tolerance to the sedative effect of ethanol are compared to mechanism underlying appetite or aversive olfactory learning and memory.

What would be okay, is to use aversive olfactory learning and memory together with the mushroom body as working model. That would required to have detailed introduction to the function of DPM and Apl neurons in different forms of memory in addition to a description what part of the MB contribute to what kind of memory. Here, an introductory figure would be great. We also know that a single exposure to ethanol changes the function of dopaminergic neurons that mediate the positive reinforcing effect of ethanol (Knabbe et al., 2022). A comparison to mechanisms underlying appetitive olfactory learning and mechanism might be more interesting. Given that aversive and appetite memory requires different groups of dopaminergic neurons, I would highlight one model (aversive or appetitive).

In general, the experiments were carried out carefully and I think the data are important enough to be published in Plos Genetics. However, I recommend major revision before publishing.

Response: We thank the reviewer for their generally positive review, their careful assessment, and for providing us with helpful perspective. We address aspects of the critiques above within the specific critiques below.

In detail:

1) Learning and memory in animal models are defined by observation. An animal learns when after a previous experience a change in behavior is observed. If the behavior change persists, it is due to the memory of the previous experience. I am not sure how the term “memory like state” relate to this. Per definition ethanol tolerance is learnt and memorized (see definition above). There is literature related to this, that has not been cited and need to be included into the introduction. That might serve for a better introduction and understanding.

Nevo and Hamon, 1995

Kalant , 1998

Bitrán M, Kalant H, 1991. Learning factor in rapid tolerance to ethanol-induced motor impairment. Pharmacology, Biochemistry, and Behavior 39, 917–922.

There are two aspects to ethanol: the direct action of the drug on the cell and the association of the resulting intoxication with the environment that might influence the behavioral outcome.

In addition, the reinforcing actions of drugs of abuse is either positive or negative depending on the concentration and duration of ethanol intoxication.

Barreto PS, Lemos T, Morato GS, 1998. NMDA-receptor antagonists block the development of rapid tolerance to ethanol in mice. Addiction Biology 3, 55–64. 10.1080/13556219872344 - DOI - PubMed

Response: We now include reference to the literature you cited above.

Specifically, we added the following to the Introduction: "Rapid tolerance was first defined in vertebrates, where most major neurotransmitters, a number of neuromodulators and hormones, as well as transcriptional regulatory mechanisms are implicated in tolerance [1–3]. The role of learning and memory in tolerance is less studied. In classical studies with rodents, an inebriating dose of ethanol was paired with a difficult coordination task: the animals performed badly for the first dose and better during a second tolerance testing dose, and the improvement was dependent on new protein synthesis [4–6]. Animals given the task only during the tolerance testing dose performed no better than the first dose animals."

We also agree that the tolerance we measure has features of learning and memory: organization, perdurance, genetics, and in some cases circuitry. We continue to call what we found "memory-like states" since we are not using explicitly associative tasks where a defined neutral cue is paired and then tested, and not everyone in the field is on board with the concept of non-associative memories. We try to steer clear of taking sides and instead describe the molecular and circuit mechanisms of the phenomena. We do not yet know if the direct action of the drug or the association, or both, are what creates these memory-like states, since these are incredibly difficult to completely separate.

Finally, to address the reviewer’s important point about valence and our focus on aversive learning and memory, we added the following to the Introduction:

“How single ethanol exposure memory-like states are encoded in the brain is not known. Here, we focus on the genes and circuitry defined for classical aversive learning and memory, because it is currently better defined in Drosophila as compared to appetitive learning and memory, and because ethanol has both aversive and appetitive properties within a single exposure [7–10].”

2) The work would benefit from background information related learning and memory and its relationship to ethanol tolerance (mouse, rats see references see above).

Response: Please see our reply to critique #1. We also added at the end of the introduction that the first exposure memory-like states use different circuits from Drosophila alcohol associative memories, where ethanol inebriation is paired with neutral cues in spaced training. We were remiss in fully separating our findings from these important and better-established forms of ethanol behavioral plasticity.

3) The work would benefit from background from molecular mechanism under ethanol tolerance (GABA, glutamate balance…).

Response: We added a brief sentence and references to guide the reader to this literature. We wish to keep the focus of our Introduction and the manuscript on Drosophila, otherwise our introduction is in danger of expanding into a full review.

The sentence is a lead-in to the tolerance/learning and memory paragraph described in critique #1.

“Rapid tolerance was first defined in vertebrates, where most major neurotransmitters, a number of neuromodulators and hormones, as well as transcriptional regulatory mechanisms are implicated in tolerance [1–3]. The role of learning and memory in tolerance is less studied.”

4) Reorganization of the data. Usually it is important to know if the controls work and then see the experimental data. Not the other way around. We also read scientific articles from left to right and not right to left.

Response: We reorganized the data in all figure panels to show controls and then experimental.

5) The statistics: there is no indication of the sample size. I am not willing to count dots.

Response: Sample size is indicated in the Supplementary Table, Column E, and includes more details on the statistical analyses.

The correction of significance using Dunett’s test does not appear correct.

This is a test for multiple comparisons with a control are also called many-to-one comparisons. That means to one control. In Figure 1D. left panel, the data are compared to one control, however in addition to each other.

Response: We stand corrected by the reviewer, thank you. We made the following changes throughout the manuscript.

1. For data that passes the Shapiro-Wilk test for residual normality and the Brown-Forsythe test equal SDs, we now use an ordinary one-way ANOVA and we compare the means of preselected columns with Holm-Šídák's multiple comparison test.

2. For data that fails the Brown-Forsythe test for equal SDs, we use Brown-Forsythe followed by Dunnett's T3 post-hoc test. Our understanding is that the latter can be used for one experimental to multiple controls. We used this post hoc in the original manuscript - we now correctly specify it as "Dunnett's T3".

3. For data that fails the Shapiro-Wilk test for residual normality, we continue to use Kruskal-Wallis followed by Dunn's post-hoc.

We updated the Methods, the Supplementary Data table, the figures, and the figure legends.

To our surprise (and substantial relief), although significance generally lessened somewhat going from Dunnett's to Holm-Šídák's, no changes in "star-level" significance were manifest. The statistics were updated for the following experiments, by figure panel:

1C right, 1D right, 2A left, 2D left, 3A', 3B', 4E left, 5A left, 6A, 6A', 6B' left, 7D' right, 8C right, 8C' left, 8C' center, 8C' right, 9A' center, S1C left, S2B right, S4A' right, S4D' left, S5B' right.

The data in 1A third panel 24h memory, there is barely any memory observed. Is the E2 different from E1?

Response: Exposure 2 uses the same ethanol concentration as Exposure 1. To test if any memory/tolerance is observed, we did a trial One sample t test and a trial Wilcoxon Signed Rank test, and found that both samples were significantly different from zero for both tests. The Wilcoxon gave lesser significance: no cold shock: P=0.0122, cold shock: P=0.0024. We do not include this analysis in the manuscript since that would test the data twice.

6) In Figure 1A, the data are presented in the correct order, first “normal” than treatment group. Please resort data presentation

Response: We reorganized the data in each figure panel to show controls and then experimental.

7) The data of figure 10 does not really fit to the story and should be removed.

Response: While we see the reviewers point, we plan to leave the findings in. Our rationales are that it provides deeper understanding of the role of the mushroom body circuitry in chronic tolerance and further distinguishes first ethanol experiences, which are both goals of the research in the manuscript.

Minors:

Title: Alcohol should be ethanol

Response: Fixed.

Introduction:

Line 67: Apl and DPM are abbreviations

Response: Fixed.

Line 74: Reference is missing.

Response: Added.

Line 76 to 77: reference is missing.

Response: Added.

Results:

Line: it is not clear how ethanol tolerance is measured. First dose how long? Second dose how long? Info is also missing in material and method section. Brief introduction how experiments were done is missing.

Response: Thank you to reviewers 1 and 3 for pointing out that we failed to provide this information.

We added the following to the Introduction:

"Rapid tolerance is operationally defined as occurring after internal ethanol concentrations are returned to baseline from the initial inebriating exposure [1,11]. Both rapid and chronic tolerance in Drosophila are due to functional changes in the nervous system and not changes in ethanol metabolism [11,12]."

This response is also provided to a similar critique by Reviewer 3:

Flies are typically exposed for about 30 min, to get controls to 75-95% sedation (for example 16/20 flies lost the righting response = 80%). For rut1, amn1, and elav>amn.IR we exposed for 30-35 min at a slightly higher ethanol vapor concentration, since these mutants were quite sedation resistant.

We added the following to the Methods: “Flies were exposed to ethanol for 30 min during the first exposure (E1) to bring controls to 75-90% sedation. In rare cases where the experimental group exhibited strongly decreased sensitivity, the ethanol vapor concentration was increased to 60% and the E1 lengthened to 30-35 min"

We added the following to the first paragraph of the Results: "Rapid tolerance is induced and measured by giving flies two ethanol exposures of identical concentration, with the second exposure (E2) beginning 4-24 hr after the start of the first (E1). The number of flies in a group of 20 genetically identical individuals that lose the righting response are counted every 6 min, and the time to 50% sedation (ST50) for the group is calculated for E1 and E2. Sensitivity is the E1 ST50, and rapid tolerance is the ST50, E2-E1."

Line 87: reference for ASM and ARM is missing

Response: Added.

Question: If amn is required for in the mushroom body for AST, how that can be connected to the GABA function (Fig5) that repress Kenyon cells for AST and NMDar1 knockdown in mb cells?

Response: The short answer is we don't know, and the data is currently difficult to reconcile into a simple model. We chose to not propose models for this reason. Here are some possible models. First, APL GABA provides tonic inhibition of the Kenyon cells to modulate amn release. Second, APL GABA is released in response to ethanol providing an acute inhibitory signal to modulate amn release. Third, the GABA-A receptor acts in different Kenyon cells than amn, thus inhibition of αβ neurons may in turn cause release of amn from the γ neurons. The same applies to NMDAR1. Fourth, amn and GABA may be independent MB mechanisms that both control AST.

As we noted in response to a similar critique from reviewer 3, "The goal of this manuscript is to map out where overlap exists between alcohol memory-like states and classical memories, and to pursue important details in future research. Because there is no simple explanation we did not provide one, to help keep the reader focused on the main objective."

Line 115 to 116 : appears redundant to Line 118 to 121

Response: Deleted the second sentence, thank you.

Line 153: awkward sentence: It also mapped…

Response: Added a subject: "Radish-dependent ethanol sensitivity may also map to the APL neurons, however decreased sensitivity was only evident with one of two Radish RNAis (Fig. 3B', S3B')."

Line 163: reference is missing for shi

Response: Added Kitamoto 2001.

Line 207: Thus, activity of the GABA ergic Apl neuron… too strong statement related to DPM neuron function. The experiment DPM, UAs-Shi in Ast was not done or GAD knock down… maybe I did not see it.

Response: The data for the DPMs is in Fig. S4D,E. We added this to the sentence to help readers.

Line 276: difficult to evaluate, increase number of N or down tune…how much N anyway?

Response: 'n' is 17, 17, & 17, as specified in the Supplementary Table, and is in line with the 'n' for other chronic tolerance experiments. We changed the sentence to state "Hence, chronic tolerance appears to not require neuronal activity in the mushroom body circuitry, in contrast to classical long-term memories."

Section of Sirt 1: I am not sure how this add up to the rest of the story, especially in the light of reference 47 showing that Sirt1 terminates Mef2 induction.

Response: It relates to the role of the mushroom body circuitry in chronic tolerance development, and so we believe that it is appropriate to keep the findings in this manuscript. Understanding if Mef2 regulation underlies the chronic tolerance suppressing role of Sirt1 in the γ lobes is an interesting future direction.

Fig2: The model in E: if the neurons are not addressed or do not have a function, they should be labeled weakly or removed. It is also not clear what the arrow and cold shock means in this context and why this is not shown vor amn in the memeory model”.

Response: We followed the reviewers suggestion and we grayed out the circuit elements that are not tested or showed no function for the findings presented in each figure.

We localized amn function to the Kenyon cells. amn knockdown blocks cold shock effects on 4 hr rapid tolerance, both brain-wide (Fig. 1C) and in the Kenyon cells (Fig. 2A). We don't know where the cold shock signal originates, but it's pathway appears to be through amn in the Kenyon cells. We thank you for the suggestion to parallel this in the memory model - cold shock and amn mutants show redundant memory deficits [13,14].

Similar comments apply also for the other cartoon in Figure 3 an there on. Too much infos in a model without relationship to text blur content of model. It might be okay in a final summary model to include all infos.

Response: Agreed and changed. Thank you.

Please change the order of the controls and the experimental groups, otherwise it is very hard to follow.

Response: Done.

Discussion:

- I missed something how molecules/brain region relate to infos known about rapid tolerance in mice.

Response: While we see the value in this, it is in practice very hard to do. Even the much better-established and more thoroughly researched classical learning and memory literature in flies attempts to draw few parallels to the mouse, especially at the level of circuitry. We need much better circuitry detail in mice, and more molecular mechanistic and behavioral understanding in flies. We did add brief statements to the introduction and cite literature reviews for those interested in attempting the comparisons.

- In addition, I think it is too simple to look at ethanol exposure as “one” behavior. For example, sedation might have different valence to flies than hyperactivity.

Response: We added a “Limitations” paragraph as the second-to-last paragraph in the discussion, and we include some of likely very complex actions of even a single sedating ethanol exposure.

The added paragraph is:

"Our findings are limited in several ways. First, ethanol as a pharmacological agent has both positive and negative valence effects even within a single exposure. Here we compare ethanol responses to negative valence classical learning and memory, because current knowledge of positive valence classical learning and memory in Drosophila is more fragmentary. Second, as ethanol dose increases during a single exposure, animals proceed through a sequence of responses that likely engage partially overlapping sets of molecules and neurons. Our endpoint analysis of tolerance to the sedating effects of ethanol captures a specific aspect of ethanol action. Third, we limit our study to the APL, DPM, and Kenyon cells, whereas the classical learning and memory circuitry includes additional inputs and outputs, as well as neurons in other regions of the brain. The role of other known sites for rapid tolerance, including in the central complex, the blood-brain barrier, and parts of the circadian clock, were also not tested [15–18]."

- Rapid tolerance requires hang function in neurons of the central complex. Homer is also required in the central complex for tolerance. How does this relate to a possible function outside of the MB for ART?

Response: We added a sentence in the above “Limitations” paragraph covering these findings. We note that the Gal4s used in the pioneering 2007 Homer study (Urizar...Davis) all also express in the mushroom bodies in addition to the central complex, which was originally missed due to the relative nonspecificity of the tools and the lower resolution of the microscopy available at the time.

**Reviewer #2:** Larnerd et al. (2024) investigates the how “memory”-like ethanol tolerance states are encoded in the fly brain. Through procedural and careful molecular and circuit analyses they identify a set of neurons and molecules necessary for different forms of ethanol tolerance and sensitivity. This manuscript provides interesting comparisons between ethanol tolerance and memory; both involve mushroom body circuitry and share interesting similarities in the timing of their involvement throughout “acquisition” and “consolidation”.

Response: We thank the reviewer for their positive overview.

Although the authors compare these forms of tolerance to memory-like states, they do not explicitly state what this association could be (what constitutes the conditioned and unconditioned stimuli?). While this is briefly mentioned in the discussion (Lines 413 – 423) it would be useful to make it clearer what this possible association could be – or if the authors believe there is no explicit association being made stating this in the text. It seems like the authors suggest that the conditioned stimulus could be the odor of ethanol. In this case, it would be interesting to test smell-blind flies (e.g. olfactory mutants or shibire-ts olfactory sensory neurons) to determine if their ability to acquire tolerance depends on their ability to smell the ethanol.

Response: We increased our speculation in the Discussion. We added a paragraph toward the end that reads:

"An important outstanding question is whether ethanol memory-like states are associative or non-associative, or even a mix thereof. Cues were not explicitly paired with the initiating ethanol exposures, and they were not used to test for tolerance. Hence, association, if occurring, is made to readily available cues in the environment, or perhaps even to interoception of the pharmacological action of ethanol. A single inebriating ethanol dose similar to that used herein to induce rapid tolerance also induces ethanol preference [19]. The ethanol preference test is performed in a different environment and at a different ethanol concentration than the initiating ethanol exposure, which ruled out many potential associative cues. Thus, at least some plasticity caused from the first ethanol exposure may be non-associative."

It is not clear if the molecular targets of interest are indeed expressed in the cells the authors target for manipulation. I would recommend the authors look to Aso et al. (2019) (Figure 3 and supplements) and the FlyCellAtlas (Li et al. 2022) to compare/confirm gene expression for those cell types and adjust the wording in the paper taking into consideration this data, for those that show no expression of the genes of interest in those cell types. i.e. gap junctions inx6 and inx7 don’t appear to be clearly expressed in APL & DPM^[3]^, which could explain why the authors did not see a phenotype for the rapid tolerance.

Response: Thank you to the reviewer for pointing this out. The Ammer...Borst paper (ref 3 of the reviewer) states "One caveat of our immunohistochemical approach is that cells that weakly express innexin proteins might fall below the detection limit. This might explain, for example, why we did not detect clear innexin expression in the mushroom body, whereas other studies have found several innexin subtypes to be involved in mushroom body function.19–21." With respect to Aso 2019, it is possible that the presence of a signal is more easily interpreted than its absence. The current evidence is for (behavior with RNAi: ASM Ann-Shyn Chiang's lab, our ethanol sensitivity data), and against (expression: the papers the reviewer provides).

We think our current findings should remain for the following reasons.

1) We observed ethanol sensitivity (but not tolerance) phenotypes for both inx6 and inx7 in DPM and APL respectively, the neurons that are predicted from the learning and memory literature. Thus, we provide evidence for their function in these neurons.

2) Although we also find the immunohistochemical data compelling, genetics can sometimes uncover roles of low-expressed genes. Time and additional experiments may resolve the differences in the literature.

However, we added the following to the Discussion: "Finally we note that, although APL:DPM gap junctions are implicated for ASM and for ethanol sensitivity, cell type-specific sequencing and immunohistology with antibodies fail to detect INX6:INX7 gap junctions in these neurons [89,90]. One possibility is that gap junctions play a developmental role that manifests in adult behavior. Alternatively, behavior may be a more sensitive readout for expression."

Minor Comments

If blocking synaptic transmission of APL reduces rapid tolerance (Fig 4A) and knock-down of GABAAR in KCs also suppresses tolerance (Fig 8B), does it make sense that suppressing GABA synthesis (Fig 4D) would enhance rapid tolerance?

Response: You are correct, the current data do not make sense (specifically 4A vs 4D), within the current scope of our understanding of the coding of tolerance. One can pose ("make up") various hypotheses. For example, the APLs are known to release octopamine in addition to GABA, and both are presumably blocked by blocking synaptic transmission. Another possibility is that the synaptic transmission block is only during the rapid tolerance experiment, whereas GABA release is constitutively reduced. The goal of this manuscript is to map out where overlap exists between alcohol memory-like states and classical memories, and to pursue important details in future research. Because there is no simple explanation we did not provide one, to help keep the reader focused on the main objective.

In Figure 1A (third and fourth panels) Why are the values for the untreated groups so different between the two panels? It looks like they have not acquired any tolerance in the 4^th^ panel despite having the same treatment as the untreated flies in the 3^rd^ panel.

Response: There are day-to-day differences in the level of tolerance, especially by 24 hours. By one sample t tests and Wilcoxon singed rank tests both groups in the fourth panel are different from zero, so tolerance is measurable and there is room to observe a potential phenotype. The Wilcoxon test that results in less significance, for different from zero: no cold shock: P=0.0122, cold shock: P=0.0024. We do not include this analysis in the manuscript since that would test the data twice.

I suggest changing the colors for rapid tolerance so that it is more easily distinguishable from chronic tolerance.

Response: This is changed, thank you for the suggestion.

Some of the “Fig X” are missing a dot between Fig and X. E.g. Line 154 (Fig 3B’).

Response: Fixed, thank you.

Figure S1 has panels with bars occluding the dots and error bars.

Response: Fixed, thank you.

(Reviewer's references)

[1] Aso, Y., Ray, R. P., Long, X., Bushey, D., Cichewicz, K., Ngo, T. T., ... & Rubin, G. M. (2019). Nitric oxide acts as a cotransmitter in a subset of dopaminergic neurons to diversify memory dynamics. *Elife*, *8*, e49257.

[2] Li, H., Janssens, J., De Waegeneer, M., Kolluru, S. S., Davie, K., Gardeux, V., ... & Wolfner, M. F. (2022). Fly Cell Atlas: A single-nucleus transcriptomic atlas of the adult fruit fly. *Science*, *375*(6584), eabk2432.

[3] Ammer, G., Vieira, R. M., Fendl, S., & Borst, A. (2022). Anatomical distribution and functional roles of electrical synapses in Drosophila. *Current Biology*, *32*(9).

**Reviewer #3:** In their manuscript entitled “Memory-like states created by the first alcohol experience are encoded into the Drosophila mushroom body learning and memory circuitry in an alcohol-specific manner,” Larnerd et al. extend their previous work on rapid and chronic teolerance states by using cold shock to further define consolidated processes underlying tolerance. They define new molecular and circuit-level events by drawing further parallels between memory states and tolerance. The work is comprehensive, novel, and welcome, as it should significantly impact and integrate how we understand rapid and chronic tolerance in Drosophila.

Response: We thank the reviewer for their positive overview.

There are minor changes that I believe will help readers:

In the methods section, please specify how long flies were expose to ethanol vapor during the first exposure. I infer this is 30 minutes, but it is not obvious and readers should not need to go to previous papers for this infomration.

Response: Flies are typically exposed for about 30 min, to get controls to 75-90% sedation (for example 16/20 flies lost the righting response = 80%). For rut1, amn1, and elav>amn.IR we exposed for 30-35 min at a slightly higher ethanol vapor concentration, since these mutants were quite sedation resistant.

We added the following to the Methods:  "Flies were exposed to ethanol for 30 min during the first exposure (E1) to bring controls to 75-90% sedation. In rare cases where the experimental group exhibited strongly decreased sensitivity, the ethanol vapor concentration was increased to 60% and the E1 lengthened to 30-35 min."

We added the following to the first paragraph of the Results: "Rapid tolerance is induced and measured by giving flies two ethanol exposures of identical concentration, with the second exposure (E2) beginning 4-24 hr after the start of the first (E1). The number of flies in a group of 20 genetically identical individuals that lose the righting response are counted every 6 min, and the time to 50% sedation (ST50) for the group is calculated for E1 and E2. Sensitivity is the E1 ST50, and rapid tolerance is the ST50, E2-E1."

The w1118 mutation was used as a control for the loss-of-function mutants. Given that white mutations have been reported to lead to defects in ethanol sensitivity (Chan et al. 2014; van der Linde, et al. 2014), the authors should address this and how it may or may not impact the analysis of the loss-of-function mutants. Since these mutant phenotypes were also supported by RNAi studies, this is not a major concern.

Response: According to Chan 2014 there is no effect of w- on ethanol sedation sensitivity in an assay that parallels ours. van der Linde 2014 found a surprisingly marked decrease in sensitivity for w-. Our experience has been the same as for Chan 2014 for sedation sensitivity and rapid tolerance with negligible or no effect of white- vs wild-type or mini-w+ flies.

In the Introduction, the authors refer to the hundreds of dopaminergic neurons that innervate the mushroom bodies (page 4, line 72). This reviewer is aware of ~125 neurons innerating the MB per hemisphere: PAMs and PPL1s (i.e., Tanaka , et al. 2008; Mao and Davis, 2009). It would be helpful if the authors were a more precise here.

Response: We changed the text to read "...twenty types of dopaminergic neurons" and added appropriate references.

The role of Sirt1 is left out of Figure 11, which otherwise summarizes the findings in the maunscript.

Response: We summarize Sirt1 in Fig 10. We kept it separate since Sirt1 is not well explored in learning and memory (perhaps we can convince someone to do this!)

------

References for this response to reviewers:

1.	Fadda F, Rossetti ZL. Chronic ethanol consumption: from neuroadaptation to neurodegeneration. Prog Neurobiol. 1998;56: 385–431. doi:10.1016/s0301-0082(98)00032-x

2.	Pietrzykowski AZ, Treistman SN. The Molecular Basis of Tolerance. Alcohol Res Health. 2008;31: 298–309.

3.	Elvig SK, McGinn MA, Smith C, Arends MA, Koob GF, Vendruscolo LF. Tolerance to alcohol: A critical yet understudied factor in alcohol addiction. Pharmacology Biochemistry and Behavior. 2021;204: 173155. doi:10.1016/j.pbb.2021.173155

4.	Bitrán M, Kalant H. Learning factor in rapid tolerance to ethanol-induced motor impairment. Pharmacol Biochem Behav. 1991;39: 917–922. doi:10.1016/0091-3057(91)90053-5

5.	Nevo I, Hamon M. Neurotransmitter and neuromodulatory mechanisms involved in alcohol abuse and alcoholism. Neurochem Int. 1995;26: 305–336; discussion 337-342. doi:10.1016/0197-0186(94)00139-l

6.	Bitrán M, Kalant H. Effect of anisomycin on the development of rapid tolerance to ethanol-induced motor impairment. Pharmacol Biochem Behav. 1993;45: 225–228. doi:10.1016/0091-3057(93)90109-7

7.	Risinger FO, Oakes RA. Dose- and conditioning trial-dependent ethanol-induced conditioned place preference in Swiss-Webster mice. Pharmacol Biochem Behav. 1996;55: 117–123. doi:10.1016/0091-3057(96)00069-x

8.	Shimizu C, Oki Y, Mitani Y, Nakamura T, Nabeshima T. Factors Affecting Ethanol-Induced Conditioned Place Preference and Locomotor Sensitization in Mice. Biol Pharm Bull. 2015;38: 1935–1945. doi:10.1248/bpb.b15-00626

9.	Uhl GR, Drgonova J, Hall FS. Curious cases: Altered dose-response relationships in addiction genetics. Pharmacol Ther. 2014;141: 335–346. doi:10.1016/j.pharmthera.2013.10.013

10.	Kaun KR, Azanchi R, Maung Z, Hirsh J, Heberlein U. A Drosophila model for alcohol reward. Nat Neurosci. 2011;14: 612–9. doi:10.1038/nn.2805

11.	Scholz H, Ramond J, Singh CM, Heberlein U. Functional ethanol tolerance in *Drosophila*. Neuron. 2000;28: 261–271.

12.	Berger KH, Heberlein U, Moore MS. Rapid and chronic: two distinct forms of ethanol tolerance in Drosophila. Alcohol Clin Exp Res. 2004;28: 1469–1480. doi:10.1097/01.alc.0000141817.15993.98

13.	Tamura T, Chiang AS, Ito N, Liu HP, Horiuchi J, Tully T, et al. Aging specifically impairs amnesiac-dependent memory in Drosophila. Neuron. 2003;40: 1003–11.

14.	Turrel O, Goguel V, Preat T. Amnesiac is required in the adult mushroom body for memory formation. J Neurosci. 2018. doi:10.1523/JNEUROSCI.0876-18.2018

15.	Lange AP, Wolf FW. Alcohol sensitivity and tolerance encoding in sleep regulatory circadian neurons in Drosophila. Addict Biol. 2023;28: e13304. doi:10.1111/adb.13304

16.	Ruppert M, Franz M, Saratsis A, Velo Escarcena L, Hendrich O, Gooi LM, et al. Hangover Links Nuclear RNA Signaling to cAMP Regulation via the Phosphodiesterase 4d Ortholog dunce. Cell Rep. 2017;18: 533–544. doi:10.1016/j.celrep.2016.12.048

17.	Urizar NL, Yang Z, Edenberg HJ, Davis RL. Drosophila homer is required in a small set of neurons including the ellipsoid body for normal ethanol sensitivity and tolerance. J Neurosci. 2007;27: 4541–4551. doi:10.1523/JNEUROSCI.0305-07.2007

18.	Parkhurst SJ, Adhikari P, Navarrete JS, Legendre A, Manansala M, Wolf FW. Perineurial Barrier Glia Physically Respond to Alcohol in an Akap200-Dependent Manner to Promote Tolerance. Cell Rep. 2018;22: 1647–1656. doi:10.1016/j.celrep.2018.01.049

19.	Peru YC de PR, Ojelade SA, Penninti PS, Dove RJ, Nye MJ, Acevedo SF, et al. Long-lasting, experience-dependent alcohol preference in Drosophila. Addict Biol. 2013. doi:10.1111/adb.12105
